# Supplementary figures and images for: Multifaceted modes of γ-tubulin complex recruitment and microtubule nucleation at mitotic centrosomes
Source: J Cell Biol. 2023 Sep 12;222(10):e202212043. doi: 10.1083/jcb.202212043 (PMC10497398; doi:10.1083/jcb.202212043)

SourceDataF1

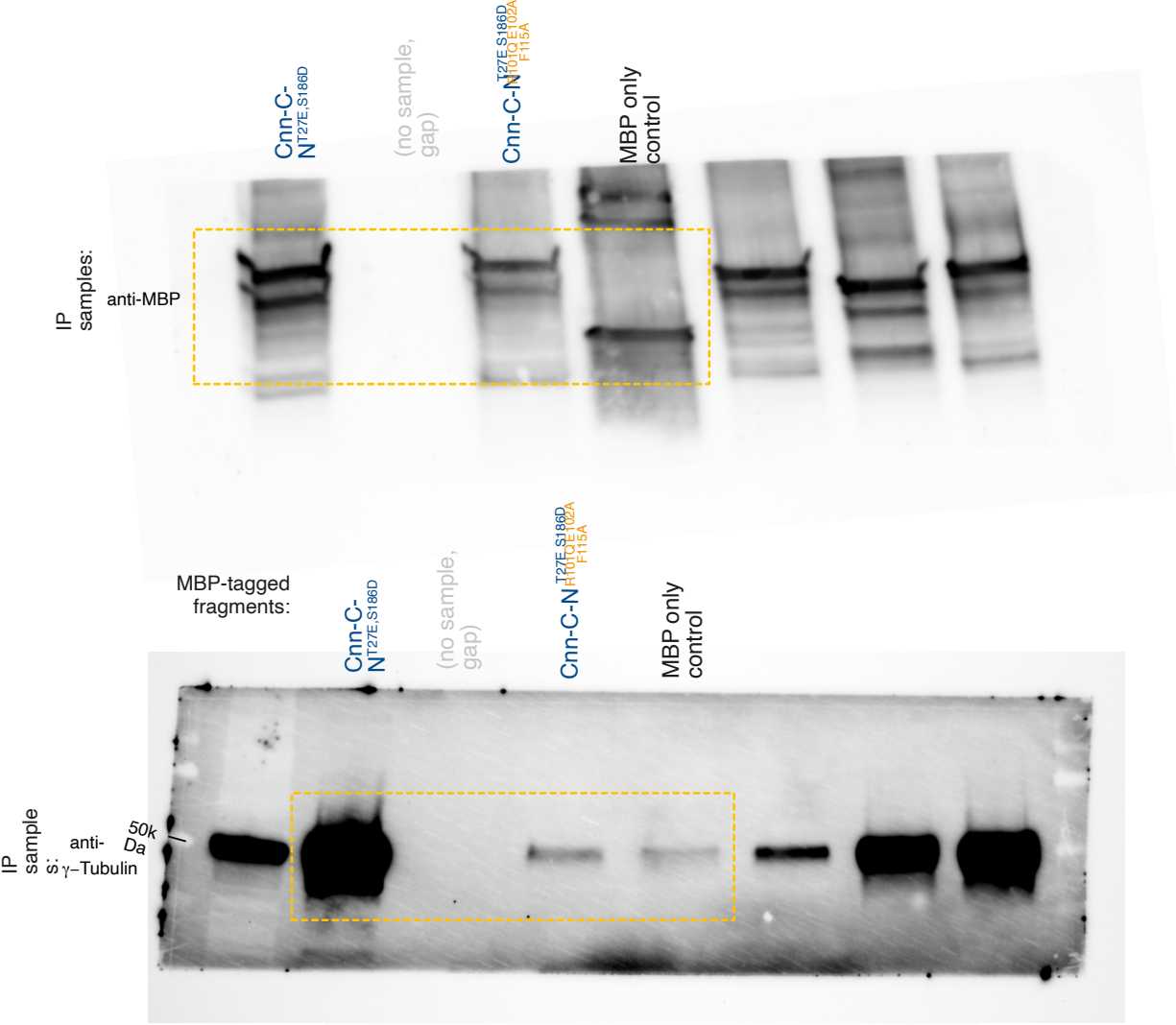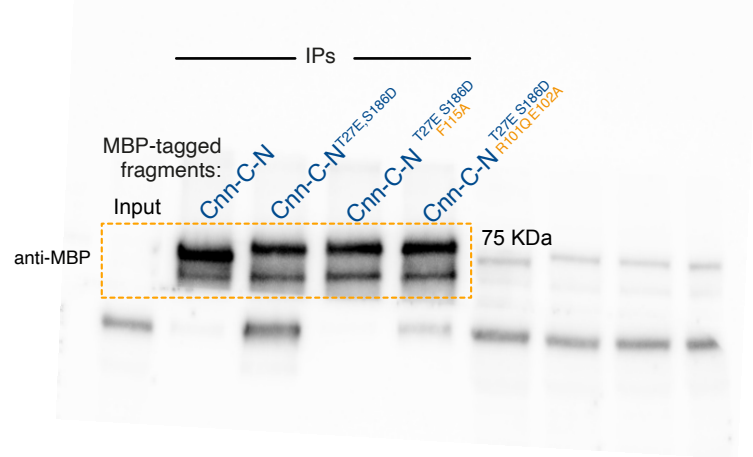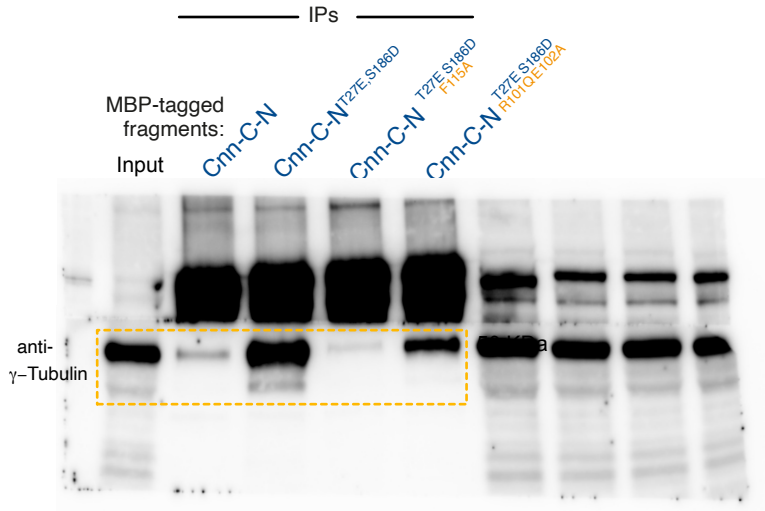

Supplement: SourceData F1 — is the source file for Fig. 1. [file JCB_202212043_SourceDataF1.pdf]

# SourceDataFS1

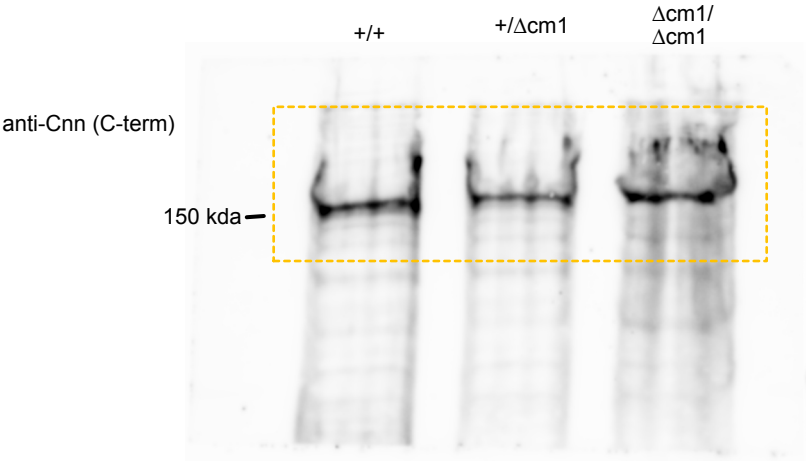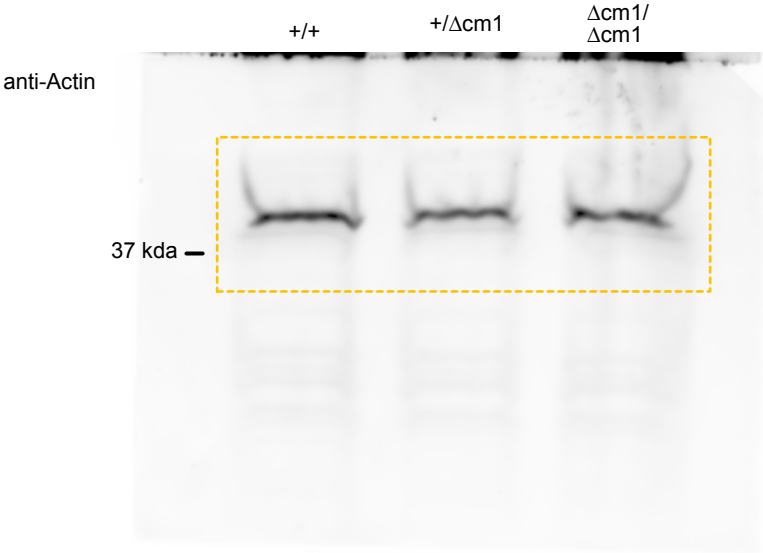

Supplement: SourceData FS1 — is the source file for Fig. S1. [file JCB_202212043_SourceDataFS1.pdf]

SourceDataFS6

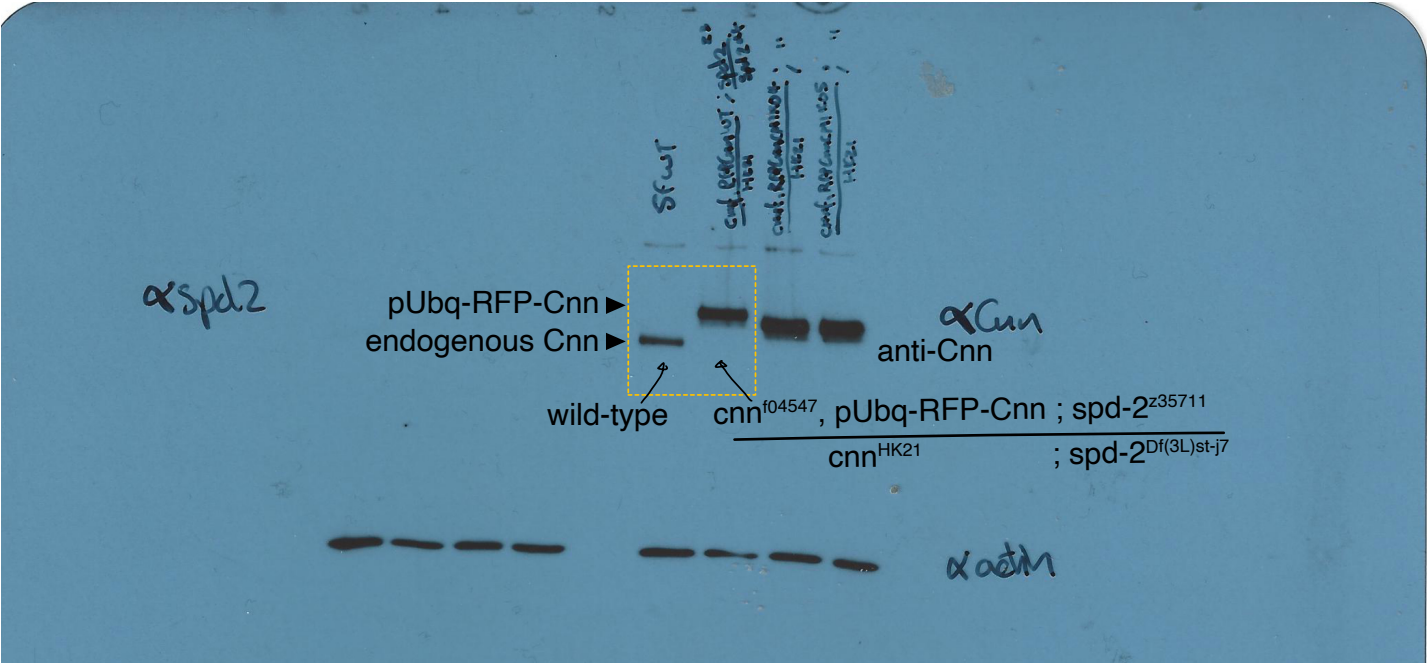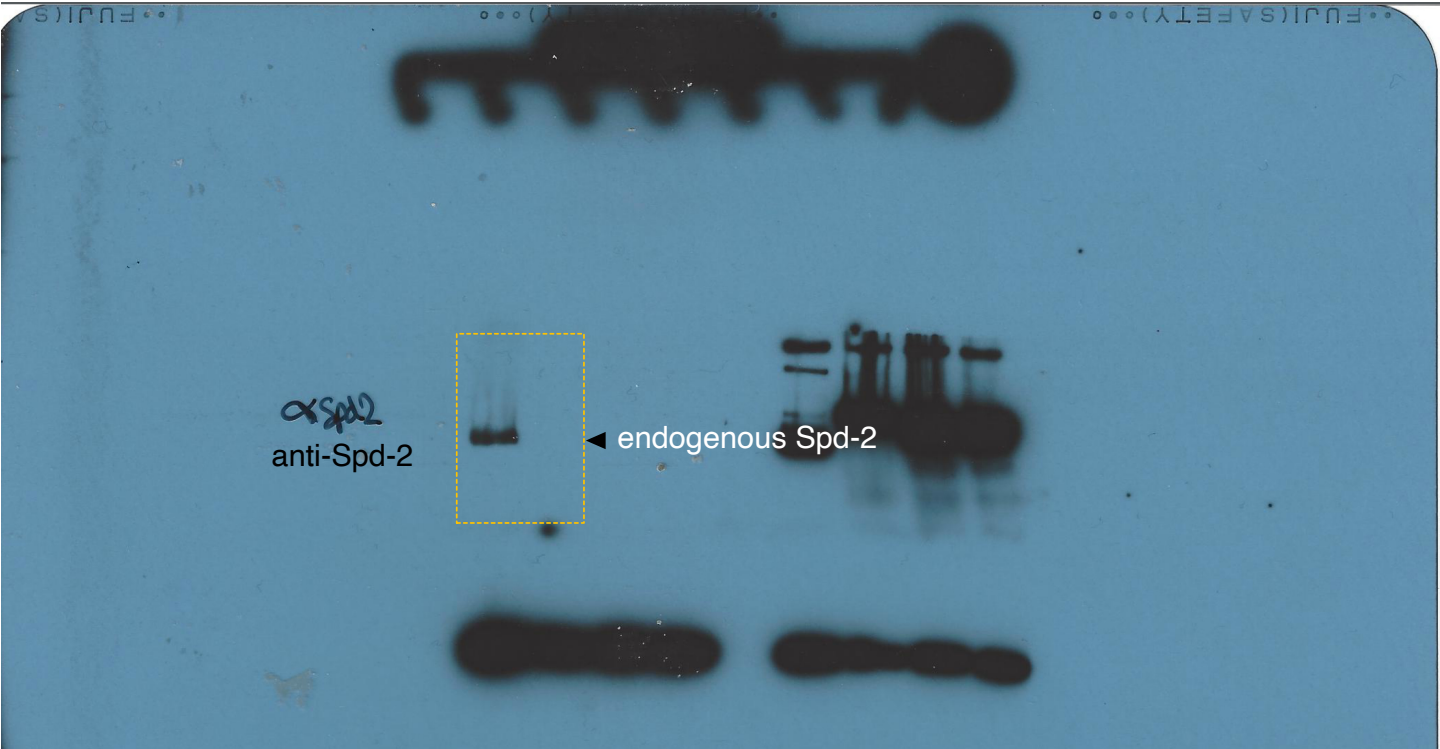

Supplement: SourceData FS6 — is the source file for Fig. S6. [file JCB_202212043_SourceDataFS6.pdf]
